# The most recent baltic sea marine hunter-gatherers? The buried individual of grave IB3 in the Suutarinniemi cemetery, Finland

**DOI:** 10.1371/journal.pone.0274953

**Published:** 2022-11-10

**Authors:** Maria Lahtinen, Ville Hakamäki, Jari-Matti Kuusela

**Affiliations:** 1 Finnish Food Authority, Helsinki, Finland; 2 Finnish Museum of Natural History, Laboratory of Chronology, University of Helsinki, Helsinki, Finland; 3 North Karelian Museum, Carelicum, Joensuu, Finland; 4 Regional Museum of Lapland, ARKTIKUM, Rovaniemi, Finland; 5 Department of Archaeology, University of Oulu, Oulu, Finland; Institucio Catalana de Recerca i Estudis Avancats, SPAIN

## Abstract

Most European hunter-gatherers slowly assimilated into farming communities during the Neolithic period. In the north these groups persisted far longer. In this paper, we present evidence from what may be one of the most recent non-agricultural sites in the region, where a marine hunter-gatherer lifestyle may have continued until as late as the 15th–16th centuries AD. The isotope composition of incremental dental analysis suggests a significant, long-term dependence on seals. This indicates that vestiges of this means of subsistence might have been present in Europe for much longer than previously thought.

## Introduction

**Fig 1:**
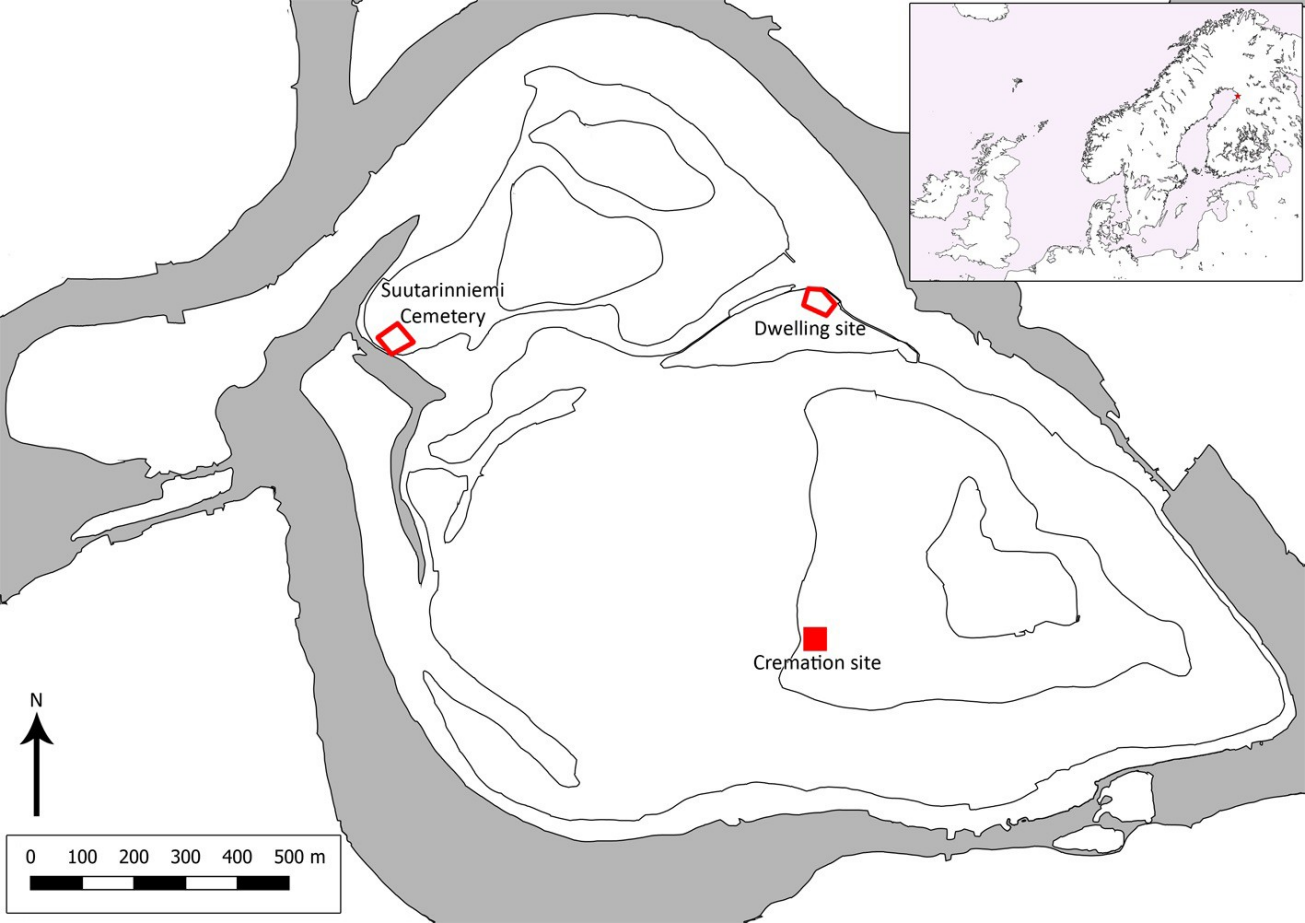
Location of the site at Suutarinniemi, a dwelling site (Pirttitörmä), and a cremation site (Kiviharju W) at Illinsaari. Our study material is from the Suutarinniemi cemetery.

Our study is focused on North Ostrobothnia in the north of Finland. This is a region on the coast of the Bothnian Bay which, in turn, is the northernmost part of the Baltic Sea (Fig 1). With low population density and a lack of centralised administration throughout most of the Middle Ages, historical sources concerned with the study region are rare prior to the 17th century AD. Coupled with a relative lack of archaeological research, this means that we know little about the region during this period. Agriculture started to spread through southern Finland during the Iron Age [1], but it is currently unclear when subsistence farming reached North Ostrobothnia. It has previously been assumed that it appeared during the Late Iron Age or Medieval period however–as will be expounded on later in the paper–this view has recently been criticised. Even after farming began in the region, hunting, fishing, and gathering remained an important part of the subsistence strategy.

In northern coastal regions, seal was a primary prey species for hunter-gatherer groups. However, with the shift away from marine hunter-gathering during the neolithisation process, the diet of the people of the Baltic transformed [2] and seal no longer dominated. After this shift seals continued to be hunted, but they no longer formed a key part of the subsistence strategy [3]. By contrast, in farming societies during the Bronze Age, the Iron Age and the historical period, the utilisation of marine resources was focused on fishing, and this was complemented by the products of farming [4–6]. Freshwater fish were utilised inland and, because the same species occupy the north Baltic Sea, freshwater lakes and rivers, the use of fish was unlikely to have been differentiated according to the catchment area. This is seen, for example, in Iin Hamina, where both freshwater and marine fish were used [4].

When exactly farming began in the northernmost Baltic Sea region is still debated. Discussions of Late Iron Age and early medieval societies, societal structures, and cultural makeup in northern Fennoscandian have, arguably, been dominated by the idea of a cultural dichotomy between those who lived on the coast and those who lived inland. The former are often seen as either direct cultural descendants of southern agricultural communities, or colonists from southern agricultural regions. The latter–either explicitly or implicitly–are seen as the forebears of present day Sámi cultures [7, 8]. It is assumed that an agricultural subsistence strategy first emerged in the northern coastal region during the Late Iron Age, perhaps as early as the 9th century AD, and that this was brought by groups who moved to the north from southern regions where agricultural practices were already deeply rooted. This conclusion was drawn primarily via toponymic research [9]. At the time this theory was presented, archaeological evidence could neither verify nor disprove it. However, since the 2010s this has gradually changed, and an expanding body of evidence has enabled a more critical assessment of this type of “colonial theory” [10, 11]. Contrary to earlier views, the available archaeological evidence points towards a more complex picture. There are, for example, distinctively local burial customs that persist until at least the 13th century, as well as signs of syncretism–i.e. a blending of existing local and newer, Christian-style burial practices–which lasted even longer [12]. This implies that interactions between immigrants and locals was primarily peaceful, and the closely-intertwined nature of these social connections meant that incoming individuals were integrated into these northern communities. Accordingly, these same studies have demonstrated that there is very little evidence to support the existence of an agricultural subsistence economy during the Late Iron Age, as relevant archaeological data–for example, farming implements–are not present [10].

Absence of evidence is not necessarily evidence of absence. On the Swedish side of the Bothnian Bay, there is some evidence to suggest that the spread of agriculture was earlier with some small traces appearing from the middle Iron Age (c. 500–800 AD) onwards [13, 14]. However it should be noted that this evidence is fairly local. Moreover, the available evidence is not indicative of widespread *subsistence* agriculture. We have no artefacts related to farming dated to this or previous periods [15], nor any other type of evidence–for example, macrofossils–which might suggest this kind of subsistence activity. Thus, on the basis of the current available evidence, we must conclude that there was no farming-based subsistence economy in the study area before the 16th century AD.

Diet is one of the key parameters signifying a change towards an agrarian society, and it is plausible to assume this also holds true in the northernmost Baltic Sea area. It can be studied using stable isotope analysis of human skeletal material as the bone protein collagen retains information regarding the main protein composition of an individual’s diet [16]. Moreover, dietary changes can be tracked at a higher resolution by studying incremental dentine samples [17]. We selected one of the most recently excavated sites in the northernmost Baltic Sea region, Suutarinniemi, for our study. Although it has no evidence of an agrarian subsistence economy [18, 19], this site is temporally close to others in the region where there is evidence for the consumption of agrarian produce [4]. This site was selected because we were interested to see if its inhabitants also consumed a diet dominated by freshwater and marine fish [4].

**Fig 2:**
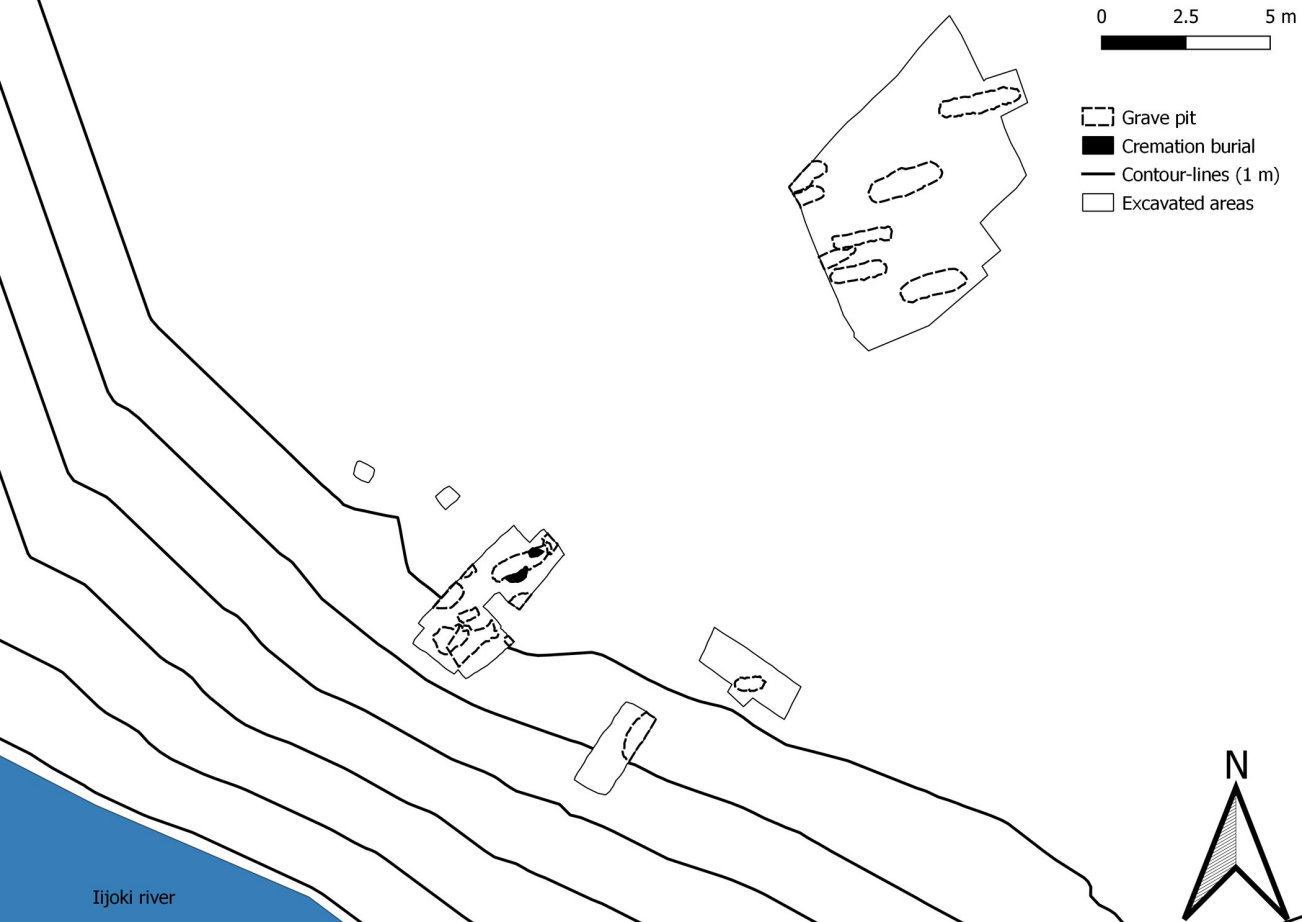
Map of the excavated parts of the Suutarinniemi cemetery.

Suutarinniemi is part of a complex of sites on an island (Illinsaari) which, when it was in use, would have been located on the shore of the Iijoki estuary. Other archaeological sites on the island (see Figs 1 and 2) include dwelling site dating to the 14th-15th century [19] and a contemporaneous cemetery [18, 20], and a slightly older cremation site dating to the 11th–12th century [21]. Only a few hundred meters to the west of the island, on the mainland, is the Iin Hamina cemetery, which was in use from the late 15th century onwards. Interestingly, analysis of the skeletal remains has revealed a diet dominated by the consumption of fish, along with some signs of agricultural products [4].

Our data is from the burial site officially named Ii Illinsaari Suutarinniemi (Finnish Heritage Agency site reference number 1000019094). Henceforth, the site will be referred to as Suutarinniemi.

Both cremations–dating to the 11th-12th centuries–and inhumations–dating primarily to the 14th–15th centuries–have been recovered from the site. However, as only uncremated bone has preserved collagen, the cremation burials could not be studied using the methods selected for this study. Thus, we analysed the isotopic composition of uncremated dentine collagen of two molars from the maxilla (second and third) of one primary inhumation; Inhumation Burial 3 (KM 40370), which will henceforth be referred to as IB3. Moreover, as we also wanted to examine whether the deceased individual was of local origin and if our results would represent local traditions, we also undertook strontium and oxygen analysis of human tooth enamel. These methods are well established within the archaeological literature (for more detail, see the supplementary material online).

A note on chronology: we utilise the Finnish chronology regarding the definition of prehistory and the Middle Ages in this paper. This clarification is necessary due to the significant variation of periodisation across Europe. Following the standard Finnish periodisation, the Late Iron Age is between the 9th century and 13th century AD, after which the Middle Ages begin. Applying the Scandinavian definition of the Middle Ages, this period is generally accepted to last until the coronation of King Gustav Vasa of Sweden in the early 16th century. For reasons of clarity and convenience of presentation, we slightly expand this definition. As such, we refer to the period of the 9th-13th centuries AD as the Late Iron Age, and the 14th-16th centuries AD as the Middle Ages.

## The Suutarinniemi cemetery

The Suutarinniemi cemetery (Fig 2) has been excavated on two occasions, first in 2013 and again in 2014. The full extent of the cemetery is still unknown, but nearly two dozen inhumation graves have been identified, eight of which have been excavated. Additionally, two cremation burials, both of which have been excavated, were discovered adjoining IB3 in 2013.

**Fig 3:**
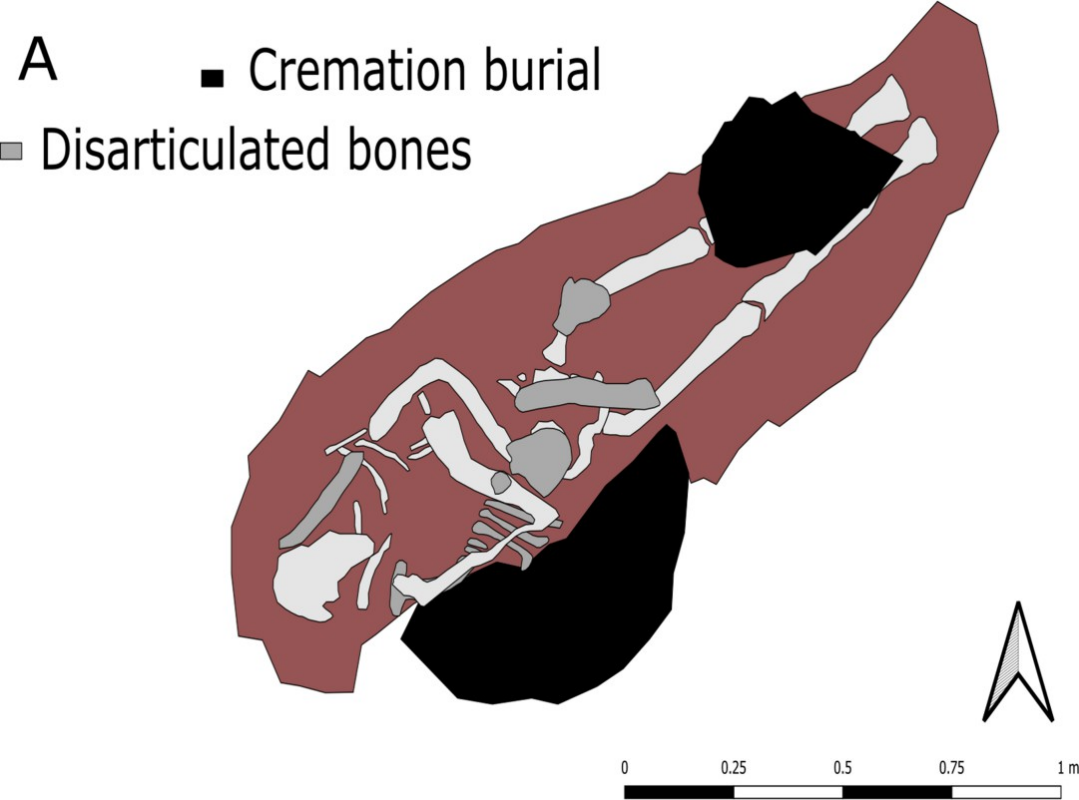
Map of burial IB3 with primary inhumation and two cremations.

IB3 is technically composed of four burials—the primary inhumation, a secondary contemporary inhumation present as several partly disarticulated human remains (see Fig 3), and two cremations [20]. It has been established that both cremations are older than either the primary or secondary inhumation burials, as they date to the 11th–12th centuries AD [20]. However, Cremation 1 was recovered from a secondary context–above the knee of the primary inhumation of IB3 –which is thought to have been interred at least a century later. Moreover, Cremation 2 had been deliberately tampered with so that roughly half of the burial remained intact, with the second half carefully distributed within the fill of IB3 [22]. The association of cremation burials with inhumation burials has also been observed in the coeval cemetery of Valmarinniemi in Keminmaa, some 65 km further north up the coast [22, 23].

The primary inhumation burial has been radiocarbon dated with a result of 610 ± 30 BP (Beta-382691), reservoir corrected to 1510–1870 AD. Typically the reservoir effect is not very strong in the northernmost Baltic Sea, and is estimated to be approximately 50 years [24, 25]. Both the archaeological and literary evidence suggest that the correction greatly exaggerates the date, so the burial most likely dates to the 15th century, with the very early 16th century being the absolute latest date with some archaeological plausibility [22]. The skeleton of the primary burial was poorly preserved, with only parts of the skull—such as the teeth–and some long bones still consisting of solid bone. Nevertheless, the shape of the skeleton was very clearly visible in the sandy soil, and although there was a lack of solid material, the shapes of individual bones and joints were still clearly observable during excavation.

The secondary deceased in the burial has been radiocarbon dated to the 14th or early 15th century AD (Ua-50696, 588 ± 36 BP). This secondary burial is present as several disarticulated human bones–some of these are above and others below the primary burial. Some of the bones had very likely still been articulated at the time of burial. Below the primary inhumation, a poorly preserved left half of a pelvic bone and part of a left half of a ribcage–consisting of four poorly preserved rib-bones–were discovered. The ribs were in a configuration suggesting they had been articulated at the time of deposition. This implies that part of the left half of a human torso had been deposited at the bottom of the grave, and that the primary inhumation had been laid on top of it. This kind of deposition–with disarticulated or partly articulated human remains in an inhumation burial–is not uncommon for the region and period. All in all, three inhumation burials in Suutarinniemi contained disarticulated human remains in addition to the primary burial, and there are several burials with the same features at the aforementioned cemetery of Valmarinniemi [12]. In contrast, however, these disarticulated bones are unlikely to be the remains of older burials as is the case at Suutarinniemi, as no older, disturbed burial pits were observed. Moreover, the placement of the bones–often a skull or a piece of one–appears to have been quite particular [12, 20, 22]. Thus, this feature is more likely to have been part of the burial ritual itself, and is perhaps an example of the syncretistic burial custom of the time which combined simple inhumations–possibly influenced by Christian traditions–with local, non-Christian practices [12].

## Materials and methods

The dentine carbon and nitrogen isotope composition, and the strontium and oxygen isotope composition, was analysed from the enamel of two teeth–the second and third upper molars (M2 and M3 respectively)–from the primary, uncremated individual in Burial IB3. Unlike bone collagen dentine does not reform. As such, it preserves the dietary signals from the time it developed, thus retaining key dietary information. Therefore it is possible to study temporal dietary history using incremental dentine analysis (see more [17, 26]). The second molar in the maxilla typically reaches its full length between 2.5 and 14.5 years of age, and the third molars in the maxilla–the wisdom teeth–are formed between the ages of 8.5 and 19.5 years [27]. However, it should be noted that these ages are estimates and that there is a fair degree of variation between individuals, especially in the case of wisdom teeth. Nevertheless, the fact that in both cases the age range is from the early childhood until early adulthood–covering at least a decade or so–means that these teeth hold information pertaining to diet over the long term, and not just over the first few years of an individual’s life.

Organic fractions in dentine are mostly collagen, a fibrous protein that is not water soluble and which is very resistant to alteration. However, post burial processes begin to decompose bone material, and diagenetic alteration can cause incorrect results in collagen studies [28–31]. This alteration can be recognised by the following quality indicator values: in (likely) unaltered collagen, the total carbon is between 28.8 and 44.8 per cent [32]; total nitrogen is between 10.2 and 16.5 per cent [32, 33]; the collagen yield is not lower than 3 per cent [32]; and the molecular ratio of nitrogen and carbon is between 2.9 and 3.5 [34]. These criteria were used to quality check every sample in this study.

Strontium samples are obtained from enamel which is mostly bioapatite, the mineral part of teeth. Strontium is water soluble, and therefore more likely to be in contact with soil after burial than collagen. However, enamel is very dense material which means that water inflow is minimal. This makes enamel very resistant to alterations and, as such, it is typically used in mobility studies. The same enamel samples were also used for oxygen isotope analysis.

The methods used in this study followed published protocols for collagen extraction [17, 35]. The teeth were cut from the middle into two halves. The half tooth was demineralised in 0.5 M HCl in the freezer. From the soft tissue, 0.5 mm horizontal slices were cut using a scalpel. Each slice–i.e each sample–was heated to 75°C for 48 hours. The samples were rotated in a centrifuge for 30 minutes and then freeze dried. Dentine carbon and nitrogen isotope composition were measured in duplicates at Bradford University with a Thermo Delta plus XL IRMS. Results were reported in delta notation (δ) per mil (‰) relative to the VPDB and AIR scale. Internal and international standards (IAEA-N1, IAEA-N2, IAEA-600, ANU Sucrose, PEF1) were used for accuracy testing, and 0.2 ‰ or greater was obtained.

Carbonate carbon and oxygen isotope compositions were analysed from the enamel. The surfaces of the samples were cleaned mechanically using a handheld drill device. The samples were powdered using a silicate mortar and pestle, and they were then washed using ion exchanged MilliQ water. The samples were analysed at Braford University using the Thermo Delta plus XL IRMS. Results were reported in delta notation (δ) per mil (‰) relative to the VPDP and VSMOW scale, respectively. Internal and International standards (IAEA-CO8, NBS19) were used for accuracy testing. Accuracy of 0.1 ‰ was obtained for carbon isotope composition, and 0.2 ‰ or greater for oxygen isotope composition.

The strontium sample surfaces were cleaned mechanically using a handheld drill device. The samples were pre-treated using strontium specific resin column chemistry. The strontium isotope composition was measured at Durham University using Thermo Fisher Neptune (MC-ICP-MS). International standard NBS-987 was used, and an accuracy of 0,00009 or greater was obtained. Fractionation in the analysis was normalised using the standard protocol [36, 37].

## Results

**Fig 4:**
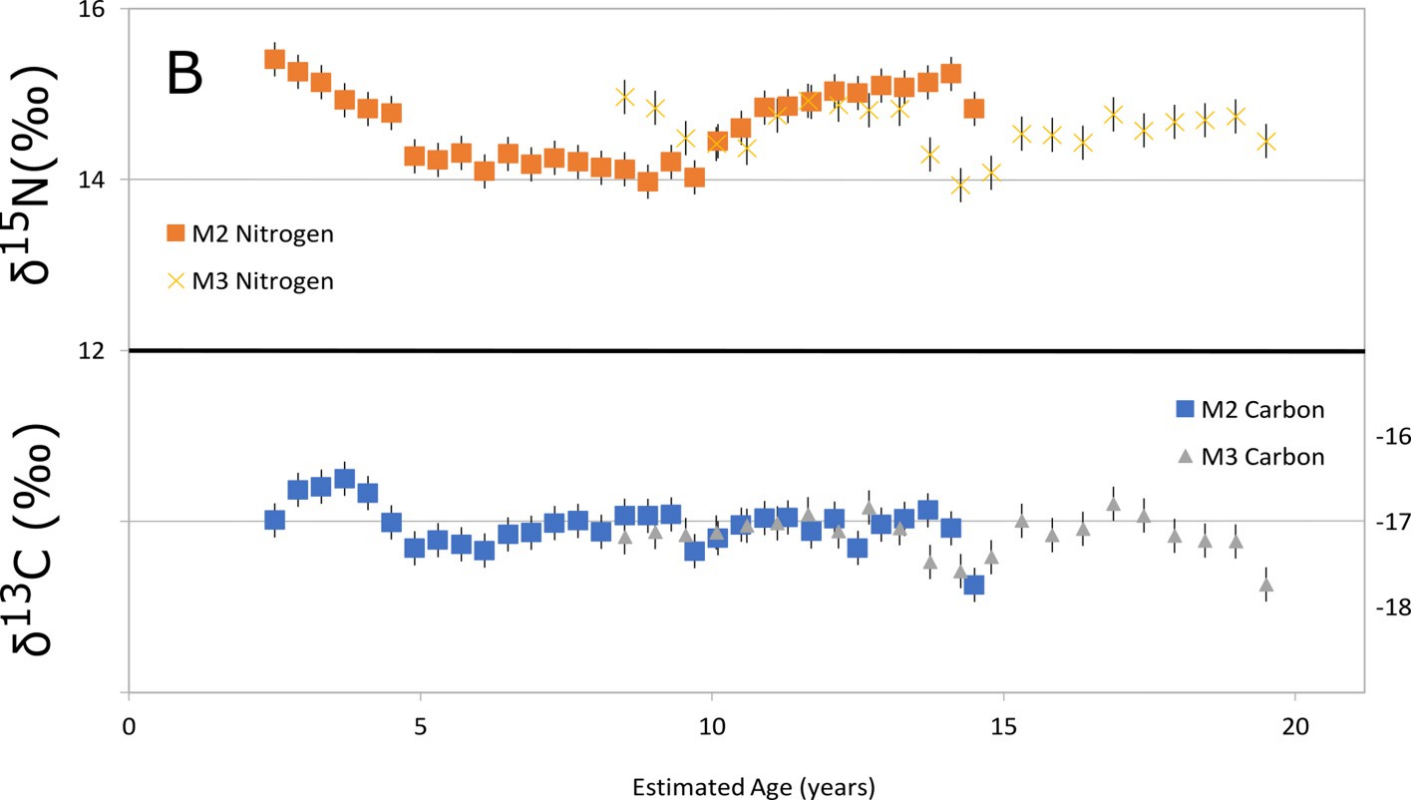
The isotope composition of incremental dentine at Suutariniemi burial IB3, in temporal resolution. The resulst have been obtained from one individual. Results show low variation and constant subsistence based on seals.

The results are reported in detail in the supplementary materials available online. The incremental analysis of the dentine collagen shows that the carbon isotope composition varied between -16.5 and -17.7 ‰, and the nitrogen isotope composition between 14.0 and 15.4 ‰ (Fig 4 and S1 File). The overall results can be described as homogeneous, and the isotope composition profiles as flat. These results represent–approximately–ages between 2.5 and 14.5 years for the second molar, and between 8.5 and 19.5 years for the wisdom tooth [27]. As such, the diet of the individual likely remained consistent and was highly dependent on seals (see Table 1 and Fig 2 in S1 File). Both the carbon and nitrogen isotope composition changed to lower carbon or nitrogen delta values during their early life, more specifically before the age of 5. This can indicate a lengthy weaning practise (see supplementary material for more discussion). A breastfed child is one step higher in trophic level than the mother, and they thus have higher nitrogen and carbon delta values relative to her isotope composition. This process is reversed when a child stars to eat solid foods, and this should be seen in the incremental dentine curves. A second change, an increase in the nitrogen isotope composition, can be seen between the age of 10 and 15. During this time, carbon isotope variation is approximate to the scale of the analytical accuracy, changing little between the age 5 and 15. An increase in nitrogen isotope composition could be something physiological, such as a lack of food or another kind of stress, but more research into the behaviour of stable isotopes in human body is needed to verify this.

Dentine carbon isotopes also fractionate from diet to tissue, at approximately to 1–3 ‰ [38]. Unfortunately our preliminary data on local animals is very limited due to poor preservation conditions and a lack of research. Nonetheless, the difference in mean local Bothnian Bay seal collagen carbon and IB3 human dentine values is approximately 1.2 ‰, which is in the range of what can be considered trophic level enrichment. As such, it further supports our interpretation that seal was a main dietary item.

The results of carbon isotope composition analysis of the tooth enamel carbonate are -11.9 and -11.7 ‰ (for more, see supplementary material). The carbonate carbon derives from all food sources including carbohydrates and lipids [38]. The results are very similar to those found in the enamel of Pitted Ware people, which are −12.1‰ to −9.8‰ [39]. In both cases, the difference is smaller than the accuracy of the measurements (±0.2‰). It has been suggested that this shows the use of terrestrial sources of food [39]. However, even if such a study uses a linear mixing model based on a brackish water area, it does not use background data from the Baltic Sea. Modern analyses show that seal fatty acids, -24.7‰ to -26.9‰, are similar in their carbon isotope composition to C3 plants [40]. Even when considering the SUESS affect–which accounts for 1.5 ‰—the values still draw the carbonate values towards those of a terrestrial diet. Osteological and archaeological evidence both suggest that the Pitted Ware culture were heavily dependent on seals as a food source, which supports the interpretation of isotope analyses on collagen as set out in this study [41].

The strontium isotope composition of the tooth enamel from IB3 are 0.719525 for the second molar and 0.712556 for the third molar (for more detail, see the supplementary information). The strontium values in the second molar can be found locally but the value in the third molar is, as was expected, skewed towards marine values [42]. The oxygen isotope composition in both teeth shows that the individual was likely a local (see supplementary information).

## Discussion

Many scholars assume that the hunter-gatherer way of life was largely over in Europe once the Neolithic period began, and farming began to take over between 8000 and 5000 BC [43]. However, in the northern subarctic regions, this was clearly a slow process. At present, there is no evidence for subsistence agriculture in the study region dating earlier to IB3 burial [20]. This is particularly evident at the Suutarinniemi cemetery, where the analysis of burial IB3 indicates that the subsistence economy continued to be centered on marine hunting. This observation is supported by the nearby dwelling site of Pirttitörmä, which has produced no evidence of farming implements or tools [19, 44].

There is a long history of exploiting marine resources in the northern latitudes. On the coast of the north eastern Baltic Sea, seal is the most common marine bone type found at Stone Age dwelling sites on the coast [45]. In the Northern Ostrobothnian region, on the Swedish and Finnish side, there is also indirect evidence for this economy dating to the Bronze Age and early Iron Age, including the presence of pits which were used for cooking seal fat [46]. Seals were extensively hunted in Swedish coastal areas throughout the Iron Age up until around 1100 AD. It has been noted that, after these locations were colonised by Swedish farming and fishing communities by 1279 AD, “…all the coastal sealing sites along the Bothnian coast had been abandoned” [47]. Our results suggest that there was a similar process on the (current) Finnish side of the Bothnian Bay (Nurmi et al. 2020) but that it was slower, with seals continuing to be exploited after they no longer were on the Swedish side.

**Fig 5:**
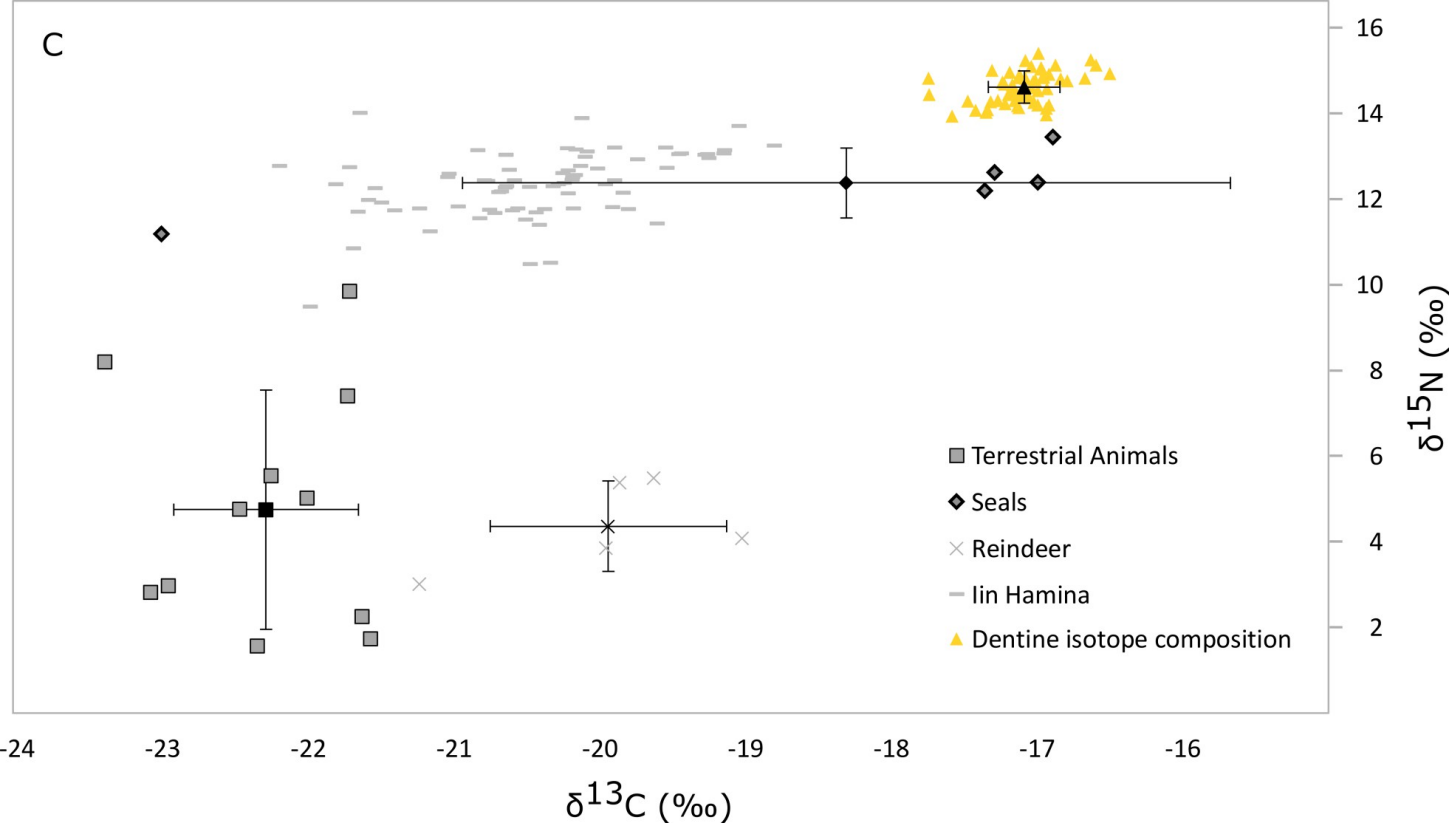
The isotope composition of the Iin Hamina bone collagen bulk samples [4] and the later, Suutarinniemi individual in IB3, in relation to local terrestrial animals, reindeer and seals. The results from Suutarinniemi are from one individual (see Fig 4).

At the moment, the archaeological evidence cannot conclusively prove that seals were the main prey item throughout the long history of the Northern Ostrobothnian area. However, since there is no evidence for intensive cultivation practices either, it is plausible to assume that the consumption of marine mammals continued until the time of the Suutarinniemi cemetery burials. As to the advent of farming in Northern Fennoscandia, agricultural implements–sickles, grinding stones, scythes, shears, plowshares etc.–are absent from the region until at least the 15th–16th century AD [10]. This is not to say that agricultural practices would have been completely absent–as noted previously, some pollen analyses from northern Sweden suggest that agriculture may have been practiced sporadically during the Iron Age [14]. However, this would have been small-scale farming, not consistent subsistence agricultural practice. This did not exist in the north this early, as there is no archaeological evidence for it on the Finnish side of the Bay [11]. The Iin Hamina site–which dates to between the late 15th and 17th centuries AD, and is close to Suutariniemi–has produced cow bones in the soil of its burial, which indicate some sort of animal husbandry [48]. However, interestingly, the diet of the Iin Hamina deceased appears to be more fish based [4, 49] (see also Fig 5) when compared to the deceased in IB3.

The results of our analyses of the human dentine isotope composition shows that the individual buried in IB3 depended on seals as a main protein source. Their diet continued unchanged throughout childhood until early adulthood, where the upper limit of our research methodology lies. This suggests that seals were the principal dietary item throughout the year, rather than being a seasonal addition. This type of diet has only been observed among hunter-gatherer groups around the Baltic Sea area [50]. However, and this is an important caveat, it is notable that our data is the diet of one individual–the primary burial of IB3. At this moment, of all the excavated burials in the Suutarinniemi cemetery, this particular person appears to be somewhat unique in general–they were the only one associated with an extensive amount of disarticulated human bones from a “secondary” burial. In all other burials where “additional” bones were present, there were only one or two. Furthermore, IB3 was the only burial at the site–at least that we currently know of–which had older cremations incorporated into it. How extensive this practice may have been at the site is, as of yet, unknown, as the majority of the cemetery remains unexcavated. Therefore, it is entirely possible that the individual in IB3 is a special case for some reason. However, even taking this caveat into account, the diet of this individual shows that this way of life–that of a marine mammal hunter–was still known to the people who buried their dead in the Suutarinniemi cemetery.

Suutarinniemi is the most recent, currently known site in the Baltic area where signs of a strongly seal-based subsistence strategy can be observed. Though seals were consistently hunted in the past beyond the 15^th^ and 16^th^ centuries, they did not form the core of any diet, even in the Northern Ostrobothnian region. Farming communities on the Baltic Sea shores continued to utilise marine resources but, instead of seals, fish became one of the key components of a diet which was complemented by farming products [4, 6]. Where sealing continued, it was a seasonal hunting practice which took part during the early spring and late autumn [3]. In the modern period seal bones continue to be found in archaeological sites, but in low numbers [51]. Similar developments are evident in the Northern Ostrobothnian region. Here, while the inhabitants of Suutarinniemi continued to rely on seals as a food source, the evidence from the later cemetery at Iin Hamina indicates that their diet was fish-based, and likely supplemented by the products of farming.

The incremental data shows that the diet of the individual buried in IB3 was dominated by seal during their childhood, which is unlikely to have been a decision that they made for themselves. This may indicate that at least one family living in the area utilised seals as their main source of protein. Given that bone preservation is very poor in northern Europe and the fact there was a continuing custom of cremation, even one find is important to our understanding of the wider context. The isotope analysis also suggests that this individual was born locally, which clearly indicates the continuation of a culture which relied on seals as a food source throughout the 15th century AD. This demonstrates how marginal–but well-connected–regions in Northern Europe can nevertheless contain significant archaeological information which deepens our understanding of the complexity of culture, the nature of cultural connectivity, and the degree to which we share a common heritage.

## Conclusions

The spread of farming was very slow across Europe. It has been previously suggested that farming was introduced very late in the northernmost latitudes and that the subsistence strategy never became one centred on agriculture. However, until now, it has not been known how long seal-based subsistence strategies were the foundation for life in this region. Our study indicates that marine hunting continued to play a vital role until the 15th or 16th century AD, and the longevity of “old” traditions should be taken into consideration when studying the Medieval period. In a similar manner, it is perhaps unsurprising that the cold, distant north continues to shock people, as some ancient traditions are upheld in the region even today.

## Supporting information

S1 File (DOCX)
