# Supplementary material for: The most recent baltic sea marine hunter-gatherers? The buried individual of grave IB3 in the Suutarinniemi cemetery, Finland
Source: PLoS One. 2022 Nov 10;17(11):e0274953. doi: 10.1371/journal.pone.0274953 (PMC9648748; doi:10.1371/journal.pone.0274953)
Supplement: S1 File — (DOCX) [file pone.0274953.s001.docx]

Supplementary materials:

**Use of isotopes in mobility and diet studies**

The use of collagen isotope composition for diet reconstructions has a long history in ecological and archaeological studies (*1*–*4*).

The main principles are: 1. carbon cycles in marine and terrestrial environments are different, which can be recognised in the fauna and flora which lived in them (*5*); 2. trophic level effect increases both δ^15^N and δ^13^C values passing from diet to consumer (*6*)⁠; 3. different phyllogenic carbon cycle of plants (the most important are C_3_ and C_4_) affects the carbon isotope composition of plants and, in turn, the whole food chain which consumes them (*7*). In Finland, however, it is very unlikely that carbon cycle C_4_ would be detected in ancient human populations as it is mainly used by plants which have adapted to hot, arid environments(*8*)⁠; 4. collagen isotope composition is mainly affected by the digested protein (*9*, *10*)⁠, although it has been suggested that other sources of nutrition can contribute up to 20% of its signal (*11*).

In addition to of these main principles, isotope composition of bone and dentine collagen is also affected also by numerous health and environmental related factors – for example, the canopy effect, malnutrition, weaning etc. – but to a lesser degree. The main point of this kind of analysis is that it allows us to estimate the main protein source in diet of the individual based on collagen isotope composition.

In Europe, this method has been used in archaeology to distinguish between consumption of marine and terrestrial resources, as both produce different carbon isotope signals (*5*). However, in the Baltic Sea area, the 'marine' signal gets weaker as we move further from the Atlantic Ocean in the south andand the the water becomes more brackish (*12*–*14*)⁠. This is visible in the total carbon isotope composition of sediments and also in species that inhabited these habitats, such as fish and seals (*15*–*17*). It is notable that brackish water resources can have different isotope compositions depending on the part of the Baltic Sea they occupy. Moreover, the carbon composition of marine animals has varied during the different chronological periods of the Baltic Sea. Therefore, any reference samples of local fauna need to originate both 1. the same period and 2. the same area (*16*)⁠. The Bothnian Bay is one of the pools of the Baltic Sea which has its own, specific carbon cycle. [Lahtinen and Salmi](https://www.zotero.org/google-docs/?h6kR2v) (*17*), for example, have published a small set of reference samples of seals from the Bothnian Bay during the pre-industrial era. Here, the composition of ‘marine’ nitrogen and carbon isotopes appears much closer to ‘terrestrial’ signals than in other marine regions.

Mobility can be studied using strontium isotope analysis (*18*). Strontium behaves similarly to calcium and can replace it in our calcified tissues⁠. The source for this is strontium is environmental, for example in water, what we eat, and in inhaled particles. Strontium isotope fractionation is minimal and not detectable with most instruments (*19*, *20*), thus food and the individuals who eats it will have approximately the same isotope composition, with no trophic level change. Therefore by analysing the composition of strontium isotopes, we are able to determine with accuracy the overall strontium isotope composition of the environment (*21*)⁠. This method can often help to establish if a person is local, but not the place from which they originated. For the purpose of this study, the biologically available strontium isotope has been established by collecting local plant samples from around the village (*22*).

The oxygen isotope composition of enamel reflects geographical variation of  δ^18^O values in drinking waters (*23*, *24*). Because of fractionation of the δ^18^O values in the human body, the analysed oxygen isotope composition is transformed to correspond to that ofthe drinking water (*24*). TheAnalysing the oxygen isotope signal is not a precise method, as the intrapopulation variation is roughly two per mille. Nonetheless, it allows us to estimate an – approximate – location for where the person lived (*25*).

**MATERIAL**

In this study, both the dentine carbon and nitrogen isotope composition, and strontium isotope ratio were studied.

Two teeth from the Grave IB3 – the second (M2) and third (M3) upper molars – were analysed. Unlike bone collagen, dentine does not reform. As such, it preserves the dietary signal from the time it developed and, therefore, the information relating to what an individual consumed. This means that it is possible to establish a temporal dietary history by using incremental dentine analysis. M2 typically reach full length between 2.5 and 14.5 years, while M3 – the wisdom teeth – are formed between 8.5 and 19.5 years (*26*)⁠. However, it should be noted that these are estimates and that, in reality, there can be significant individual variation, especially in the case of wisdom teeth.

Organic fractions in dentine are mostly collagen. Collagen is a fibrous protein, is not water soluble and is very resistant to alteration. However, post burial processes start to decompose bone material and this diagenetic alteration can cause incorrect results in collagen studies (*27*–*30*). However, this alteration can be recognised by the following quality indicator values in (likely) unaltered collagen: total carbon percentage is between 28.8% and 44.8% (*31*)⁠; total nitrogen is between 10.2% and 16.5% (*31*, *32*); collagen yield is not lower than 3% (*31*)⁠; nitrogen and carbon molecular ratio is between 2.9 and 3.5 (*33*). These criteria were used to quality check of every sample in this study.

Strontium samples are obtained from enamel, which is bioapatite, mineral part of teeth. Strontium is water soluble, and therefore more likely to be in contact with soil after burial than collagen. However, enamel is a very tight material and water inflow is minimal. This makes enamel very resistant to alteration. Enamel was also used in this study for oxygen isotope analysis.


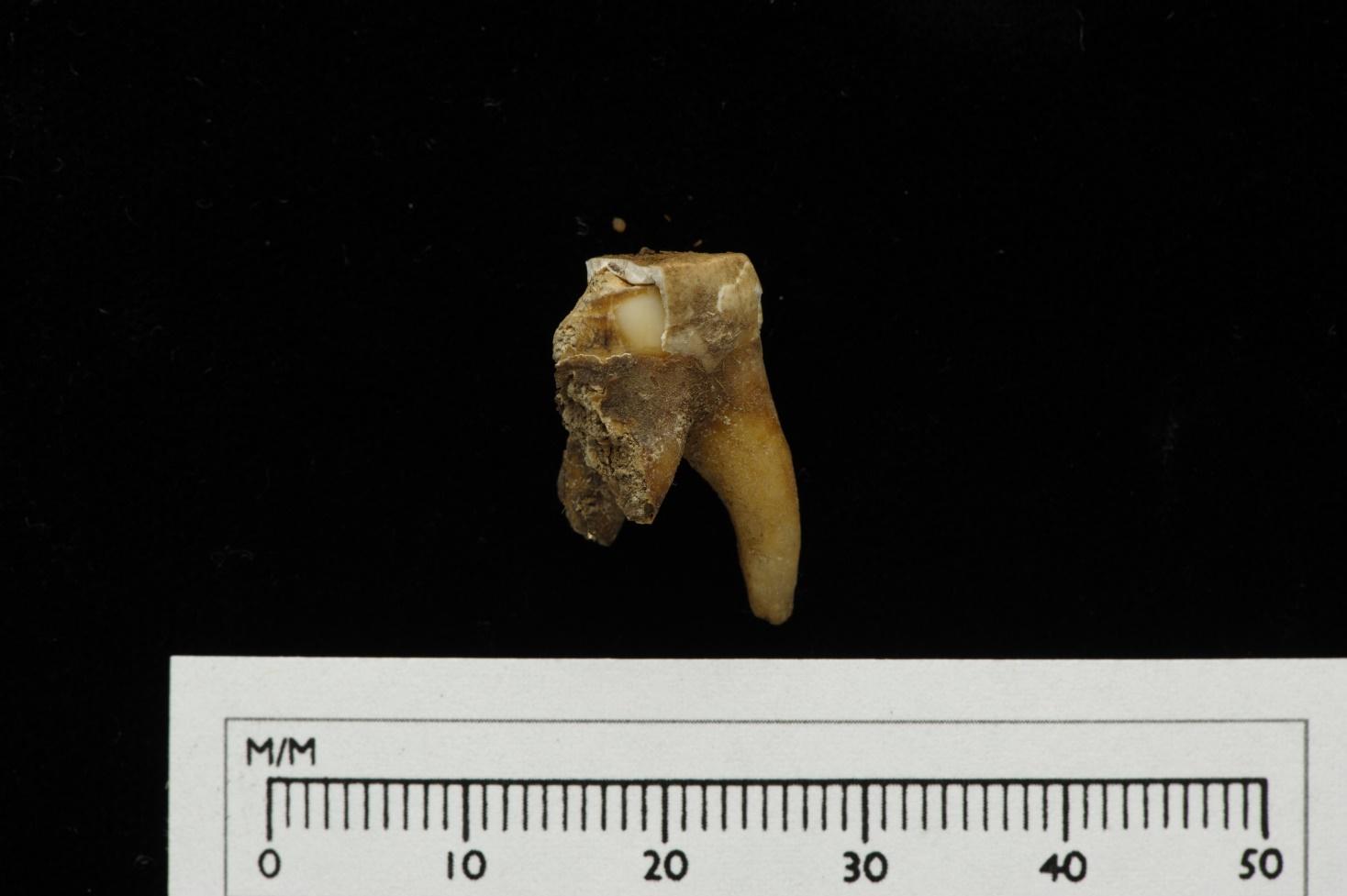


Figure 1. Tooth M2 from grave IB3. The fully-developed root was sampled.


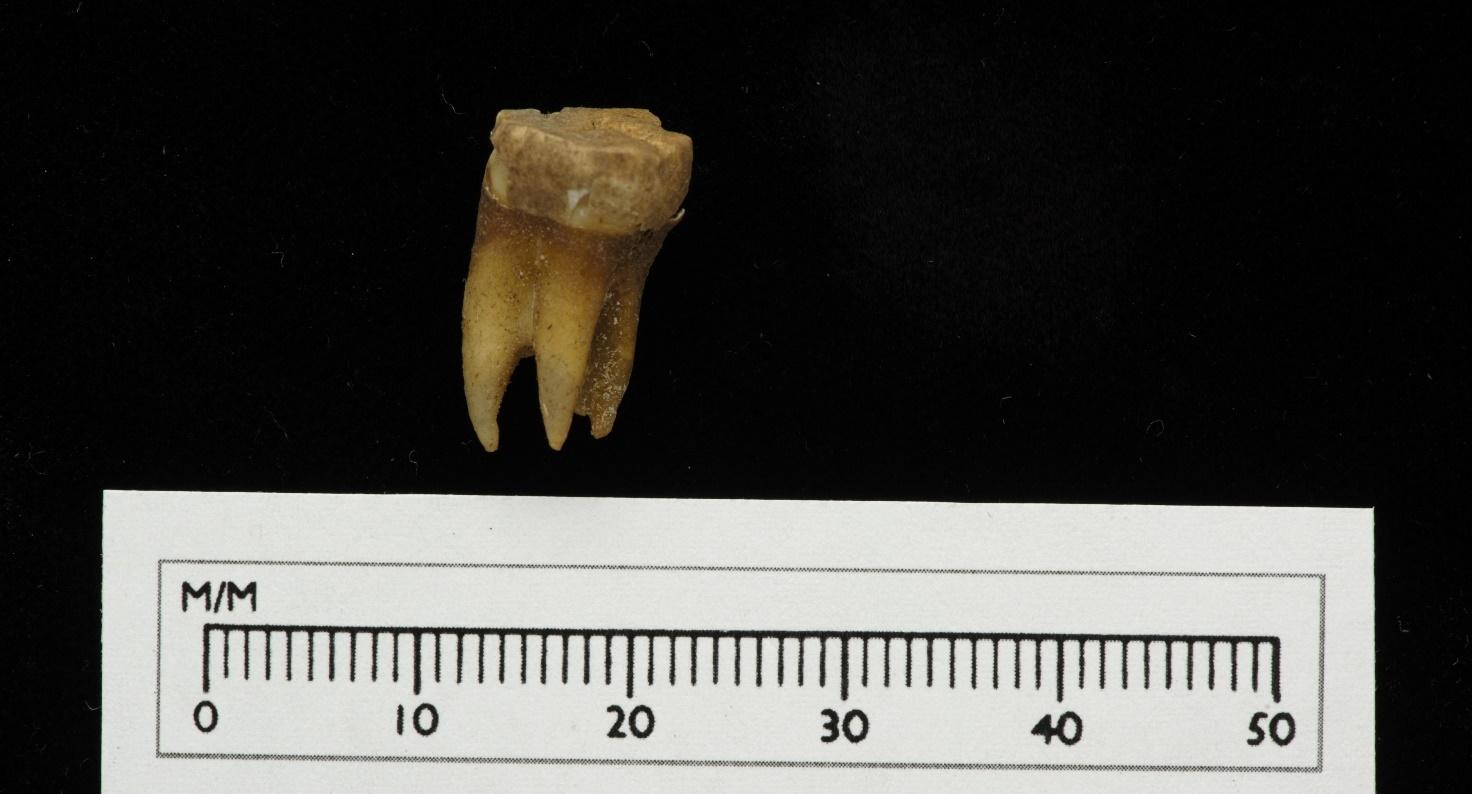


Figure 3. Tooth M3 from grave IB3.

**METHODS**

The methods used followed those set out in published protocols and collagen extractions (*34*, *35*)⁠. The tooth was cut from the middle in two halves. Each two one halves was demineralised in 0.5 M HCl in the freezer. From the softened tissue, 0.5 mm horizontal slices were cut using a scalpel. Each slice (i.e. each sample) was heated to 75 °C for 48 hours. The samples were rotated within a centrifuge for 30 minutes and freeze dried. Dentine carbon and nitrogen isotope compositions were measured in duplicates at Bradford University with a Thermo Delta plus XL IRMS. Results were reported in delta notation (δ) per mille (‰) relative to the VPDB and AIR scale. Internal and international standards (IAEA-N1, IAEA-N2, IAEA-600, ANU Sucrose, PEF1) were used for accuracy testing, and 0.2 ‰ or greater was obtained.

Carbonate carbon and oxygen isotope composition was established by analysing enamel. The surface of the enamel samples were cleaned mechanically using a handheld drill device. These were then powdered using a silicate mortar and pestle. Samples were washed using ion exchanged MilliQ water. The samples were analysed using the Thermo Delta plus XL IRMS. Results were reported in delta notation (δ) per mil (‰) relative to the VPDP and VSMOW scale respectively. Internal and International standards (IAEA-CO8, NBS19) ware used for accuracy testing, with 0.1 ‰ obtained for carbon isotope composition, and for oxygen isotope composition 0.2 ‰ or greater for oxygen isotope composition.

The surfaces of the strontium samples were cleaned mechanically using a hand held drill device. Samples were pre-treated using strontium specific resin column chemistry. The strontium isotope composition was measured at Durham University using Thermo Fisher Neptune (MC-ICP-MS). International standard NBS-987 was used for accuracy testing, with 0,00009 or greater obtained. Fractionation in the analysis was normalised using standard protocol (*36*, *37*).

Results:

**Dentine carbon and nitrogen**

All samples fulfilled the quality control indicators and are considered well preserved. Isotope composition, nitrogen and carbon content, and nitrogen and carbon atomic ratios are presented in Tables 1 and 2, and Figures 2 in the article.

| Sample ID | δ ^15^N (‰) | Mean δ ^15^N a and b (‰) | N % | δ ^13^C (‰) | Mean δ ^13^C a and b (‰) | C % | C/N | Age (years) |
| --- | --- | --- | --- | --- | --- | --- | --- | --- |
|  |  |  |  |  |  |  |  |  |
|  |  |  |  |  |  |  |  |  |
| ML3-M2-1a | 15.4 | 15.4 | +15.1 | –16.85 | –17.0 | 41.8 | 3.23 | 2.5 |
| ML3-M2-1b | 15.42 |  | +15.4 | –17.12 |  | 41.7 | 3.17 |  |
| ML3–M2-2a | 15.23 | 15.3 | +15.4 | –16.52 | –16.6 | 42.1 | 3.19 | 2.9 |
| ML3-M2-2b | 15.29 |  | +15.6 | –16.73 |  | 42.3 | 3.16 |  |
| ML3-M2-3a | 15.16 | 15.1 | +15.6 | –16.47 | –16.6 | 42.5 | 3.18 | 3.3 |
| ML3-M2-3b | 15.12 |  | +15.4 | –16.71 |  | 41.7 | 3.16 |  |
| ML3-M2-4a | 14.90 | 15.0 | +15.3 | –16.51 | –16.5 | 41.7 | 3.19 | 3.7 |
| ML3-M2-4b | 14.96 |  | +15.4 | –16.49 |  | 41.6 | 3.14 |  |
| ML3-M2-5a | 14.81 | 14.8 | +15.4 | –16.63 | –16.7 | 42.0 | 3.18 | 4.1 |
| ML3-M2-5b | 14.84 |  | +15.4 | –16.70 |  | 41.5 | 3.13 |  |
| ML3-M2-6a | 14.75 | 14.8 | +15.8 | –16.89 | –17.0 | 42.8 | 3.16 | 4.5 |
| ML3-M2-6b | 14.81 |  | +15.6 | –17.13 |  | 42.1 | 3.14 |  |
| ML3-M2-7a | 13.90 | 14.3 | +15.6 | –17.64 | –17.3 | 43.1 | 3.21 | 4.9 |
| ML3-M2-7b | 14.65 |  | +15.9 | –16.99 |  | 42.8 | 3.14 |  |
| ML3-M2-8a | 14.23 | 14.2 | +15.7 | –17.11 | –17.2 | 42.5 | 3.16 | 5.3 |
| ML3-M2-8b | 14.23 |  | +15.8 | –17.33 |  | 42.6 | 3.15 |  |
| ML3-M2-9a | 14.29 | 14.3 | +15.5 | –17.33 | –17.3 | 42.0 | 3.17 | 5.7 |
| ML3-M2-9b | 14.34 |  | +15.7 | –17.21 |  | 42.1 | 3.13 |  |
| ML3-M2-10a | 14.07 | 14.1 | +15.8 | –17.25 | –17.3 | 42.6 | 3.16 | 6.1 |
| ML3-M2-10b | 14.13 |  | +15.6 | –17.43 |  | 42.0 | 3.14 |  |
| ML3-M2-11a | 14.35 | 14.3 | +15.6 | –17.11 | –17.2 | 42.3 | 3.17 | 6.5 |
| ML3-M2-11b | 14.25 |  | +15.5 | –17.20 |  | 41.9 | 3.15 |  |
| ML3-M2-12a | 14.21 | 14.2 | +15.4 | –17.13 | –17.1 | 41.9 | 3.18 | 6.9 |
| ML3-M2-12b | 14.15 |  | +14.3 | –17.13 |  | 38.6 | 3.14 |  |
| ML3-M2-13a | 14.25 | 14.3 | +15.4 | –16.98 | –17.0 | 41.9 | 3.17 | 7.3 |
| ML3-M2-13b | 14.26 |  | +17.1 | –17.05 |  | 46.3 | 3.15 |  |
| ML3-M2-14a | 14.23 | 14.2 | +15.6 | –17.07 | –17.0 | 42.3 | 3.17 | 7.7 |
| ML3-M2-14b | 14.19 |  | +12.9 | –16.92 |  | 34.8 | 3.14 |  |
| ML3-M2-15a | 14.09 | 14.1 | +15.3 | –17.33 | –17.1 | 41.6 | 3.18 | 8.1 |
| ML3-M2-15b | 14.19 |  | +12.9 | –16.92 |  | 34.8 | 3.14 |  |
| ML3-M2-16a | 14.00 | 14.1 | +15.7 | –16.97 | –16.9 | 42.4 | 3.16 | 8.5 |
| ML3-M2-16b | 14.24 |  | +15.1 | –16.89 |  | 41.3 | 3.18 |  |
| ML3-M2-17a | 13.97 | 14.0 | +15.4 | –16.99 | –16.9 | 42.0 | 3.18 | 8.9 |
| ML3-M2-17b | 13.98 |  | +15.6 | –16.88 |  | 42.7 | 3.18 |  |
| ML3-M2-18a | 14.18 | 14.2 | +15.4 | –16.95 | –16.9 | 41.8 | 3.18 | 9.3 |
| ML3-M2-18b | 14.23 |  | +15.1 | –16.88 |  | 41.4 | 3.19 |  |
| ML3-M2-19a | 13.86 | 14.0 | +15.2 | –17.61 | –17.4 | 42.0 | 3.21 | 9.7 |
| ML3-M2-19b | 14.19 |  | +15.3 | –17.09 |  | 41.8 | 3.19 |  |
| ML3-M2-20a | 14.42 | 14.5 | +15.3 | –17.29 | –17.2 | 42.0 | 3.20 | 10.1 |
| ML3-M2-20b | 14.48 |  | +15.2 | –17.10 |  | 41.6 | 3.19 |  |
| ML3-M2-21a | 14.57 | 14.6 | +15.7 | –17.02 | –17.0 | 42.8 | 3.18 | 10.5 |
| ML3-M2-21b | 14.64 |  | +15.3 | –17.06 |  | 41.9 | 3.19 |  |
| ML3-M2-22a | 14.76 | 14.8 | +15.5 | –17.04 | –17.0 | 42.2 | 3.18 | 10.9 |
| ML3-M2-22b | 14.93 |  | +15.2 | –16.88 |  | 42.0 | 3.22 |  |
| ML3-M2-23a | 14.74 | 14.9 | +15.2 | –16.98 | –17.0 | 41.6 | 3.19 | 11.3 |
| ML3-M2-23b | 14.99 |  | +15.2 | –16.92 |  | 41.6 | 3.20 |  |
| ML3-M2-24a | 14.86 | 14.9 | +15.3 | –17.12 | –17.1 | 41.8 | 3.19 | 11.7 |
| ML3-M2-24b | 14.95 |  | +15.2 | –17.12 |  | 42.0 | 3.22 |  |
| ML3-M2-25a | 15.03 | 15.0 | +15.5 | –16.96 | –17.0 | 42.2 | 3.18 | 12.1 |
| ML3-M2-25b | 15.04 |  | +15.7 | –16.97 |  | 42.8 | 3.18 |  |
| ML3-M2-26a | 14.98 | 15.0 | +15.3 | –17.42 | –17.3 | 42.6 | 3.25 | 12.5 |
| ML3-M2-26b | 15.05 |  | +15.3 | –17.20 |  | 41.9 | 3.21 |  |
| ML3-M2-27a | 15.08 | 15.1 | +15.3 | –17.16 | –17.0 | 41.7 | 3.17 | 12.9 |
| ML3-M2-27b | 15.12 |  | +15.3 | –16.91 |  | 41.9 | 3.19 |  |
| ML3-M2-28a | 15.04 | 15.1 | +15.2 | –16.93 | –17.0 | 41.6 | 3.18 | 13.3 |
| ML3-M2-28b | 15.11 |  | +15.2 | –17.01 |  | 41.9 | 3.21 |  |
| ML3-M2-29a | 15.11 | 15.1 | +15.3 | –16.77 | –16.9 | 41.8 | 3.18 | 13.7 |
| ML3-M2-29b | 15.17 |  | +15.2 | –16.97 |  | 42.0 | 3.21 |  |
| ML3-M2-30a | 15.23 | 15.2 | +15.3 | –17.15 | –17.1 | 41.8 | 3.19 | 14.1 |
| ML3-M2-30b | 15.24 |  | +15.2 | –17.01 |  | 41.8 | 3.20 |  |
| ML3-M2-32a | 14.80 | 14.8 | +15.2 | –17.74 | –17.7 | 41.8 | 3.20 | 14.5 |
| ML3-M2-32b | 14.85 |  | +15.1 | –17.75 |  | 42.0 | 3.23 |  |
| ML3-M3-1a | 15.01 | 15.0 | +15.1 | –17.16 | –17.2 | 41.8 | 3.22 | 8.5 |
| ML3-M3-1b | 14.93 |  | +15.0 | –17.21 |  | 41.5 | 3.22 |  |
| ML3-M3-2a | 14.92 | 14.8 | +15.1 | –17.04 | –17.1 | 41.6 | 3.22 | 9.0 |
| ML3-M3-2b | 14.76 |  | +14.7 | –17.20 |  | 41.2 | 3.27 |  |
| ML3-M3-3a | 14.50 | 14.5 | +15.0 | –17.21 | –17.2 | 41.4 | 3.21 | 9.6 |
| ML3-M3-3b | 14.47 |  | +15.3 | –17.11 |  | 41.9 | 3.19 |  |
| ML3-M3-4a | 14.38 | 14.4 | +15.2 | –17.16 | –17.1 | 42.0 | 3.21 | 10.1 |
| ML3-M3-4b | 14.45 |  | +15.2 | –17.10 |  | 41.4 | 3.17 |  |
| ML3-M3-5a | 14.29 | 14.4 | +15.0 | –17.09 | –17.0 | 41.2 | 3.21 | 10.6 |
| ML3-M3-5b | 14.45 |  | +15.3 | –17.01 |  | 41.7 | 3.19 |  |
| ML3-M3-6a | 14.74 | 14.8 | +15.0 | –17.12 | –17.0 | 41.3 | 3.21 | 11.1 |
| ML3-M3-6b | 14.76 |  | +14.2 | –16.93 |  | 38.8 | 3.19 |  |
| ML3-M3-7a | 14.85 | 14.9 | +15.4 | –16.99 | –16.9 | 42.2 | 3.20 | 11.6 |
| ML3-M3-7b | 14.99 |  | +15.1 | –16.84 |  | 41.4 | 3.20 |  |
| ML3-M3-8a | 14.91 | 14.9 | +15.1 | –16.93 | –17.1 | 41.4 | 3.19 | 12.2 |
| ML3-M3-8b | 14.84 |  | +16.0 | –17.30 |  | 44.7 | 3.25 |  |
| ML3-M3-9a | 14.83 | 14.8 | +15.3 | –16.90 | –16.8 | 42.0 | 3.19 | 12.7 |
| ML3-M3-9b | 14.80 |  | +13.4 | –16.77 |  | 36.8 | 3.19 |  |
| ML3-M3-10a | 14.80 | 14.8 | +15.2 | –17.06 | –17.1 | 41.7 | 3.19 | 13.2 |
| ML3-M3-10b | 14.86 |  | +16.8 | –17.10 |  | 46.0 | 3.19 |  |
| ML3-M3-11a | 14.33 | 14.3 | +15.1 | –17.48 | –17.5 | 41.5 | 3.20 | 13.7 |
| ML3-M3-11b | 14.26 |  | +14.5 | –17.46 |  | 39.6 | 3.19 |  |
| ML3-M3-12a | 13.87 | 13.9 | +15.5 | –17.45 | –17.6 | 42.4 | 3.19 | 14.3 |
| ML3-M3-12b | 14.00 |  | +13.2 | –17.71 |  | 36.1 | 3.20 |  |
| ML3-M3-13a | 14.06 | 14.1 | +15.5 | –17.36 | –17.4 | 42.5 | 3.20 | 14.8 |
| ML3-M3-13b | 14.10 |  | +19.2 | –17.47 |  | 52.1 | 3.17 |  |
| ML3-M3-14a | 14.46 | 14.5 | +15.3 | –17.09 | –17.0 | 42.1 | 3.20 | 15.3 |
| ML3-M3-15a | 14.62 |  | +15.2 | –16.90 |  | 41.8 | 3.20 |  |
| ML3-M3-15b | 14.42 | 14.5 | +21.0 | –17.19 | –17.2 | 57.1 | 3.18 | 15.8 |
| ML3-M3-15b | 14.63 |  | +14.8 | –17.13 |  | 40.4 | 3.18 |  |
| ML3-M3-16a | 14.46 | 14.4 | +15.0 | –17.13 | –17.1 | 41.5 | 3.22 | 16.4 |
| ML3-M3-16b | 14.40 |  | +11.8 | –17.04 |  | 32.6 | 3.23 |  |
| ML3-M3-17a | 14.79 | 14.8 | +15.5 | –16.81 | –16.8 | 42.6 | 3.20 | 16.9 |
| ML3-M3-17b | 14.74 |  | +21.7 | –16.77 |  | 59.0 | 3.17 |  |
| ML3-M3-18a | 14.60 | 14.6 | +15.3 | –16.99 | –16.9 | 41.9 | 3.20 | 17.4 |
| ML3-M3-18b | 14.56 |  | +15.1 | –16.87 |  | 41.0 | 3.17 |  |
| ML3-M3-19a | 14.67 | 14.7 | +15.3 | –17.08 | –17.2 | 42.1 | 3.22 | 17.9 |
| ML3-M3-19b | 14.68 |  | +17.3 | –17.25 |  | 47.0 | 3.17 |  |
| ML3-M3-20a | 14.74 | 14.7 | 15.4 | –17.23 | –17.2 | 42.4 | 3.22 | 18.5 |
| ML3-M3-20b | 14.65 |  | 14.1 | –17.21 |  | 38.4 | 3.18 |  |
| ML3-M3-21a | 14.73 | 14.7 | 15.2 | –17.23 | –17.2 | 42.1 | 3.22 | 19.0 |
| ML3-M3-21b | 14.75 |  | 15.4 | –17.23 |  | 42.2 | 3.20 |  |
| ML3-M3-22a | 14.51 | 14.5 | 15.2 | –17.95 | –17.7 | 42.1 | 3.24 | 19.5 |
| ML3-M3-22b | 14.39 |  | 15.3 | –17.53 |  | 41.7 | 3.19 |  |

Table 1. Sample results for the δ^15^N value, N%, δ^13^C value, C%, molecular ratio and estimated age of the sample. Mean values have been calculated from duplicates a and b.

|  | MIN δ^15^N | MAX δ^15^N | MIN δ^13^C | MAX δ^13^C | Range δ^15^N | Range δ^13^C |
| --- | --- | --- | --- | --- | --- | --- |
| M2 | 14.0 | 15.4 | -17.7 | -16.5 | 1.4 | -1.3 |
| M3 | 13.9 | 15.0 | -17.7 | -16.8 | 1.0 | -0.9 |

Table 2 Summary of the nitrogen and carbon isotope composition and ranges for both molars.


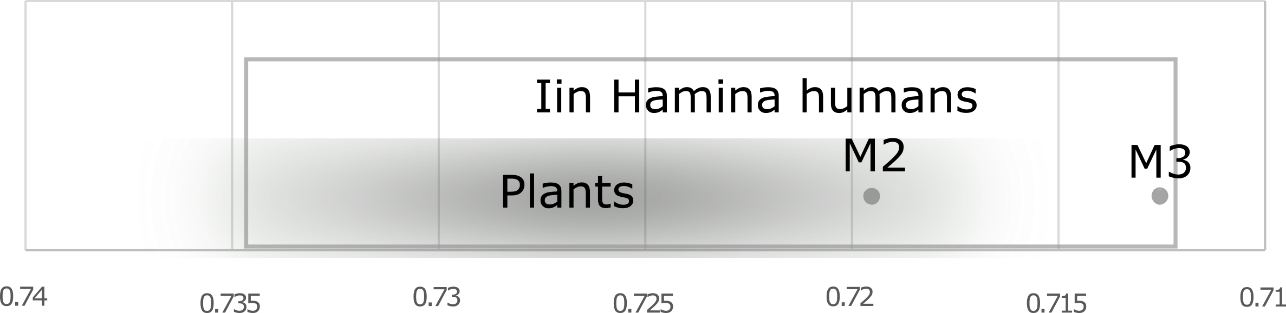


Figure 3. Strontium isotope composition of humans buried in the Iin Hamina cemetery, plant samples (n=16) collected around the village, and the tooth enamel (n=12) analysed in this study (*22*).

Typically, the fractionation from the diet to eater has been proposed to be 3-6‰ for nitrogen isotope composition (*38*). However, this is a simplified figure and there is likely to be a difference depending on whether the body needs to modify the protein parts by chemical reactions (non-essential amino acids), or if it can be used as it is (essential amino acids). Not all protein is used or even digested equally. When there are more selective reactions, fractionation from food to tissue is likely to be bigger. Controlled feeding experiments have proposed that the quality of the protein is a significant factor in the fractionation rate (*39*). To measure the protein absorption between different proteins, the field of nutritional science uses the term ´protein quality´ to reflect how well it is digested and how much it essential amino acids it contains. Seal meat has excellent protein composition for a human diet and very high in essential amino acids (*40*). Thus, it is reasonable to assume that fractionation would be ina the lower end of the scale.

It is evident that the individual in Grave IB3 has – approximately – a three per mil difference in the nitrogen composition compared to seals living in the Bothnian Bayduring the pre-industrial era in the Bothnian Bay. A similar three per mille difference between seal collagen and human collagen has been observed in Scandinavian populations consuming a seal dominated diet (*41*, *42*). This low trophic level shift is likely due to the fact that both seal fat (blubber) and meat contain high amounts of essential amino acids (*40*), which requires oflittle synthesis and therefore results in less fractionation from diet to tissue⁠. This is supported by the results of controlled feeding experiments, where it has been estimated that this type of biologically high value foodstuff leads to a smaller trophic level effect (*39*)⁠.⁠ This would suggest that seals, and notably not fish, were the main prey species for the individual buried at the Illisaari site. Fish in the Northern Ostrobothnian area are known to have a lower nitrogen isotope composition, and a fish dominated diet would result in a lower nitrogen isotope composition in human remains (*17*).

The results of this study indicate a constant use of marine protein during the early life of the individual buried in Grave IB3. The profiles on the dentine incremental isotope composition can be described as flat. This indicates little variation in diet from young age through to early adulthood, and therefore suggests a reliance on consistent subsistence strategies. The isotope composition of M3 cluster together inon a scatter plot, and the range – the difference between the smallest and greatest value – of the carbon and nitrogen isotope composition is approximately one per mil. The range is slightly greater for M2.

A decreasing nitrogen and carbon isotope composition can indicate the end of weaning period (*43*). The dentine profiles of IB3 show slightly elevated nitrogen isotope compositions during the early life. This is likely to have caused the larger range and the poorer correlation ofin the profile of M2. If these higher values are due to weaning, it can be suggested that this continued until the individual was of approximately 4 years old. From roughly the age of 10 until 14, the carbon and nitrogen isotope composition increases.

**Strontium isotope composition**

| Sample | **87Sr/86Sr** | 2SE | **87Sr/86Sr normalised** | 2SE |
| --- | --- | --- | --- | --- |
| **ILLI-3-M2** | 0.719556 | **0.000011** | **0.719525** | **0.0000105** |
| **ILLI-3-M3** | 0.712587 | **0.000012** | **0.712556** | **0.0000121** |

Table 3. Strontium isotope composition of the teeth enamels.

Normalised strontium isotope composition varies from 0.7126 to 0.7195. In previous studies, the local plant samples had a second sigma range of 0.7163 – 0.7347 (*22*). The Bothnian Bay strontium isotope composition is 0.709572 (±0.000024) and the River Ii is 0.7305 (*44*).

The two different teeth from Grave IB3 have visibly different strontium isotope compositions. It is currently is unknown how large any intra population difference would be in this environment would be, however the range of plants analysed have a larger range, which suggests that this is a possible factor.

| Sample | Weight | δ^13^C_VPDB_ (‰) |  | δ^18^O_VSMOW_ (‰) |  | δ^18^O_DW_ (‰) Daux 4 | δ^18^O_DW_ (‰) Daux 6 |  |
| --- | --- | --- | --- | --- | --- | --- | --- | --- |
| ILLI-3-M3 | 2.335 | -11.9 |  | 21.3 |  | -15.9 | -14.7 |  |
| ILLI-3-M2 | 2.71 | -11.7 |  | 21.4 |  | -15.9 | -14.7 |  |

Table xx. Oxygen δ^18^O_VSMOW_ ‰ values and δ^13^C_VPDB_ values (‰).

Table 4. The sample carbonate carbon and oxygen isotope delta values, and estimated oxygen delta values for rain water (based(*24*) using (*45*) equations 4 and 6).

The oxygen isotope composition analysed from the tooth sample was used to calculate an estimate of the isotope composition of drinking water (δ^18^O_DW_) that the individual drank during their lifetime. TheBoth teeth from IB3 produced exactly same results, transforming into -15.9 and -14.7‰ depending on which conversion formula was used.

During the time the individual from the Iin Illinsaari burial was alive, the climate was colder than it is today (*46*).This period was part of the cold climate anomaly called the Little Ice Age, which possibly started earlier in North Finland than in Central Europe. During this phenomenon, “the coldest temperatures were observed for Finland during the 1251–1350 period (0.8 °C below the 20th century level)”(*46*). The evidence from this study supports this, with the annual temperature approximately one degree lower compared to the modern reference data from nearbyis Rovaniemi. Modern ground water samples from Rovaniemi produce δ^18^O values suggesting that annual variation is between -14.0 and -14.8‰ (*47*), while the andmean of the surface waters is -14.2‰ [(*48*)](https://www.zotero.org/google-docs/?duOXzZ). This corresponds well with the drinking water values obtained for the purpose of this analysis.

When combining the two analyses, and considering both the oxygen and strontium isotope composition of the tooth enamel, the resultant values are considered local. Correspondingly, this indicates that the individual in IB3 was from the area.

References:

1. B. N. Smith, S. Epstein, Biogeochemistry of the Stable Isotopes of Hydrogen and Carbon in Salt Marsh Biotal, 738–742 (1970).

2. E. T. Degens, M. Behrendt, B. Gotthardt, E. Reppmann, Metabolic fractionation of carbon isotopes in marine plankton--If. Data on samples collected off the coasts of Peru and Ecuador. *Deep-Sea Res.*, 10 (1968).

3. M. DeNiro, S. Epstein, Influence of diet on the distribution of carbon isotopes in animals. *Geochim. Cosmochim. Acta*. **42**, 495 (1978).

4. M. DeNiro, M. Schoeniger, Stable carbon and nitrogen isotope ratios of bone collagen: variations within individuals, between sexes, and within populations raised on monotonous diets. *J. Archaeol. Sci.* **10**, 199–203 (1983).

5. B. S. Chisholm, D. E. Nelson, H. P. Schwarcz, Stable-Carbon Isotope Ratios as a Measure of Marine Versus Terrestrial Protein in Ancient Diets. *Science*. **216**, 1131–1132 (1982).

6. M. Schoeninger, Trophic Level Effects on 15N/14N and 13C/12C Ratios in Bone Collagen and Strontium Levels in Bone Mineral. *J. Hum. Evol.* **14**, 515–525 (1985).

7. U. Gowik, P. Westhoff, The Path from C _3_ to C _4_ Photosynthesis. *Plant Physiol.* **155**, 56–63 (2011).

8. J. R. Brooks, L. B. Flanagan, N. Buchmann, J. R. Ehleringer, Carbon isotope composition of boreal plants: functional grouping of life forms. *Oecologia*. **110**, 301–311 (1997).

9. S. H. Ambrose, L. Norr, in *Prehistoric Human Bone* (1993), pp. 1–37.

10. S. Jim, V. Jones, S. H. Ambrose, R. P. Evershed, Quantifying dietary macronutrient sources of carbon for bone collagen biosynthesis using natural abundance stable carbon isotope analysis. *Br. J. Nutr.* **95**, 1055 (2007).

11. R. Fernandes, M.-J. Nadeau, P. M. Grootes, Macronutrient-based model for dietary carbon routing in bone collagen and bioapatite. *Archaeol. Anthropol. Sci.* **4**, 291–301 (2012).

12. K.-C. Emeis, U. Struck, T. Blanz, A. Kohly, M. Voß, Salinity changes in the central Baltic Sea (NW Europe) over the last 10 000 years. *The Holocene*. **13**, 411–421 (2003).

13. B. Deutch, U. Berth, Differentiation of western and eastern Baltic Sea cod stocks (Gadus morhua) by means of stable isotope ratios in muscles and otoliths. *J Appl Ichthyol*. **22**, 538–539 (2006).

14. D. C. Orton, D. Makowiecki, T. de Roo, C. Johnstone, J. Harland, L. Jonsson, D. Heinrich, I. B. Enghoff, L. Lõugas, W. Van Neer, A. Ervynck, A. K. Hufthammer, C. Amundsen, A. K. G. Jones, A. Locker, S. Hamilton-Dyer, P. Pope, B. R. MacKenzie, M. Richards, T. C. O’Connell, J. H. Barrett, Stable isotope evidence for late medieval (14th-15th C) origins of the eastern Baltic cod (Gadus morhua) fishery. *PloS One*. **6**, e27568 (2011).

15. J. H. Barrett, D. Orton, C. Johnstone, J. Harland, W. Van Neer, A. Ervynck, C. Roberts, A. Locker, C. Amundsen, I. B. Enghoff, S. Hamilton-Dyer, D. Heinrich, A. K. Hufthammer, A. K. G. Jones, L. Jonsson, D. Makowiecki, P. Pope, T. C. O’Connell, T. de Roo, M. Richards, Interpreting the expansion of sea fishing in medieval Europe using stable isotope analysis of archaeological cod bones. *J. Archaeol. Sci.* **38**, 1516–1524 (2011).

16. P. Ukkonen, K. Aaris-Sørensen, L. Arppe, L. Daugnora, A. Halkka, L. Lõugas, M. J. Oinonen, M. Pilot, J. Storå, An Arctic seal in temperate waters: History of the ringed seal ( *Pusa hispida* ) in the Baltic Sea and its adaptation to the changing environment. *The Holocene*. **24**, 1694–1706 (2014).

17. M. Lahtinen, A.-K. Salmi, Mixed Livelihood Society in Iin Hamina – a Case Study of Medieval Diet in the Northern Ostrobothnia, Finland. *Environ. Archaeol.* **24**, 1–14 (2019).

18. J. Ericson, Strontium isotope characterization in the study of prehistoric human ecology. *J. Hum. Evol.* **14**, 503–514 (1985).

19. J. Fietzke, a. Eisenhauer, *Geochem. Geophys. Geosystems*, in press, doi:10.1029/2006GC001243.

20. L. Halicz, I. Segal, N. Fruchter, M. Stein, B. Lazar, Strontium stable isotopes fractionate in the soil environments? *Earth Planet. Sci. Lett.* **272**, 406–411 (2008).

21. R. Capo, B. Stewart, O. Chadwick, Strontium isotopes as tracers of ecosystem processes: theory and methods. *Geoderma*. **82**, 197–225 (1998).

22. M. Lahtinen, L. Arppe, G. Nowell, Source of strontium in archaeological mobility studies—marine diet contribution to the isotopic composition. *Archaeol. Anthropol. Sci.* **13**, 1 (2020).

23. A. Longinelli, Oxygen isotopes in mammal bone phosphate: A new tool for paleohydrological and paleoclimatological research? *Geochim. Cosmochim. Acta*. **48**, 385–390 (1984).

24. C. a Chenery, V. Pashley, A. L. Lamb, H. J. Sloane, J. a Evans, The oxygen isotope relationship between the phosphate and structural carbonate fractions of human bioapatite. *Rapid Commun. Mass Spectrom. RCM*. **26**, 309–19 (2012).

25. E. Lightfoot, T. C. O’Connell, On the Use of Biomineral Oxygen Isotope Data to Identify Human Migrants in the Archaeological Record: Intra-Sample Variation, Statistical Methods and Geographical Considerations. *PLOS ONE*. **11**, e0153850 (2016).

26. S. J. AlQahtani, M. P. Hector, H. M. Liversidge, Brief communication: The London atlas of human tooth development and eruption. *Am. J. Phys. Anthropol.* **142**, 481–490 (2010).

27. F. D. Pate, J. T. Hutton, The use of soil chemistry data to address post-mortem diagenesis in bone mineral. *J. Archaeol. Sci.* **15**, 729–739 (1988).

28. T. D. Price, J. Blitz, J. Burton, J. A. Ezzo, Diagenesis in prehistoric bone: Problems and solutions. *J. Archaeol. Sci.* **19**, 513–529 (1992).

29. B. K. Nelson, M. J. Deniro, M. J. Schoeninger, D. J. De Paolo, P. E. Hare, Effects of diagenesis on strontium, carbon, nitrogen and oxygen concentration and isotopic composition of bone. *Geochim. Cosmochim. Acta*. **50**, 1941–1949 (1986).

30. P. L. Koch, in *Stable isotopes in ecology and environmental science*, R. Michener, K. Lajtha, Eds. (Blackwell Scien- tific Publications, ed. 2nd, 2007), pp. 99–154.

31. S. H. Ambrose, Preparation and characterization of bone and tooth collagen for isotopic analysis. *J. Archaeol. Sci.* **17**, 431–451 (1990).

32. G. J. Van Klinken, Bone Collagen Quality Indicators for Palaeodietary and Radiocarbon Measurements. *J. Archaeol. Sci.* **26**, 687–695 (1999).

33. M. Deniro, M. Schoeninger, C. Hastorf, Effect of heating on the stable carbon and nitrogen isotope ratios of bone collagen. *J. Archaeol. Sci.* **12**, 1–7 (1985).

34. J. Beaumont, A. Gledhill, J. Montgomery, Isotope analysis of incremental human dentine: towards higher temporal resolution. **8**, 12 (2014).

35. T. Brown, D. Nelson, J. Vogel, J. Southon, Improved collagen extraction by modified Longin method. *Radiocarbon*. **30**, 171–177 (1988).

36. A. O. Nier, The Isotopic Constitution of Strontium, Barium, Bismuth, Thallium and Mercury. *Phys. Rev.* **54**, 275–278 (1938).

37. R. H. Steiger, E. Jäger, Subcommission on geochronology: Convention on the use of decay constants in geo- and cosmochronology. *Earth Planet. Sci. Lett.* **36**, 359–362 (1977).

38. T. C. O’Connell, C. J. Kneale, N. Tasevska, G. G. C. Kuhnle, The diet-body offset in human nitrogen isotopic values: a controlled dietary study. *Am. J. Phys. Anthropol.* **149**, 426–34 (2012).

39. C. T. Robbins, L. A. Felicetti, M. Sponheimer, The effect of dietary protein quality on nitrogen isotope discrimination in mammals and birds. *Oecologia*. **144**, 534–540 (2005).

40. L. A. Brunborg, K. Julshamn, R. Nortvedt, L. Frøyland, Nutritional composition of blubber and meat of hooded seal (Cystophora cristata) and harp seal (Phagophilus groenlandicus) from Greenland. *Food Chem.* **96**, 524–531 (2006).

41. G. Eriksson, Part-time farmers or hard-core sealers? Västerbjers studied by means of stable isotope analysis. *J. Anthropol. Archaeol.* **23**, 135–162 (2004).

42. E. Fornander, G. Eriksson, K. Lidén, Wild at heart: Approaching Pitted Ware identity, economy and cosmology through stable isotopes in skeletal material from the Neolithic site Korsnäs in Eastern Central Sweden. *J. Anthropol. Archaeol.* **27**, 281–297 (2008).

43. J. W. Eerkens, A. G. Berget, E. J. Bartelink, Estimating weaning and early childhood diet from serial micro-samples of dentin collagen. *J. Archaeol. Sci.* **38**, 3101–3111 (2011).

44. P. S. Andersson, G. J. Wasserburg, J. Ingri, The sources and transport of Sr and Nd isotopes in the Baltic Sea. *Earth Planet. Sci. Lett.* **113**, 459–472 (1992).

45. V. Daux, C. Lécuyer, M.-A. Héran, R. Amiot, L. Simon, F. Fourel, F. Martineau, N. Lynnerup, H. Reychler, G. Escarguel, Oxygen isotope fractionation between human phosphate and water revisited. *J. Hum. Evol.* **55**, 1138–1147 (2008).

46. S. Helama, An Overview of Climate Variability in Finland During the Common Era, 43.

47. N. Kortelainen, Isotopic composition of oxygen and hydrogen in Finnish groundwaters: Tables of groundwater monitoring in. *Geol. Surv. Finl. Rep.* **2007**, 13.

48. N. Kortelainen, thesis, Geological Survey of Finland, Espoo (2007).
